# Post-Graduate Study

**Published:** 1887-04

**Authors:** C. N. Johnson

**Affiliations:** Chicago, Ill.


### POST-GRADUATE STUDY.

BY C. N. JOHNSON, L. D. S., D. D. S., CHICAGO, ILL.

The fact that we can never become truly scientific men as the result of a collegiate education alone, leads to the question as to which is the best method to pursue in a system of post-graduate study. Heretofore, those men who have risen to high rank in the profession have done so mostly through individual effort. They have accomplished their results by long hours of labor in their own chemical or histological laboratories, with the silent help of the microscope. They have seldom had congenial spirits by their side, who were searching after the same truths and who could lend their advice or assistance in the solution of difficult problems. When a man is studying entirely by himself, the tendency is for his theories to begin to run in a groove. He allows his attention to be drawn to certain phenomena in a given case, sometimes to the exclusion of others which may bear as great a relation to the case in hand, and in this way his opinions become biased. He may even wrongly interpret the microscope in some of its minor manifestations, and thus construct a theory on a false basis. He is subject to an infinite number of mistakes, which would not be so liable to occur if he had the assistance of a man who was as zealous as he in the pursuit of knowledge.

On no other ground can we account for the fact that our most learned men will get up in a society and disagree on some fundamental principle, that should have been long ago settled by proper methods of study. If a man had a companion, or companions, in his study, who would work in harmony with him, each one would tend to keep the other from going very far in the wrong direction. More good will be accomplished in the way of post-graduate study if several members of the profession will form a class here and there, and work together for the general advancement of science. Of course this could only be done in cities, and even then there are many drawbacks to such a scheme. The work would necessarily have to be done mostly at night, and original investigation in science requires a great amount of time. It would be difficult to get a body of men who would willingly spend the time, and who would work in sufficient harmony to obtain the best results. Every member of the class would have to sink his individual interests, and each work for the common cause. They would all have to be above personal ambition, and the class should be conducted as much as possible without politics. No one member should seek glorification in the eyes of the profession. This is the greatest drawback to the attainment of pure scientific knowledge in the profession today. There is too much of a clamoring after office in all our organizations. There are too many political bosses in dentistry. If a dozen of our average dentists should organize for any purpose to-day, the probabilities are that, in four or five years, eleven of them would be scrambling for the presidency, and the other one would be wire-pulling for some favorite member. This is all wrong, and it is because it is wrong that I have presumed to mention it. It is contrary to the true spirit of professional dignity. Just so soon as members of the profession can lay aside their ambition to obtain office, and concentrate their efforts towards the attainment of knowledge, just so soon will we have a progressive profession, and render ourselves worthy of being called professional men. It is true that to the average man the mere attainment of knowledge forms a poor incentive to continued application, but I have almost come to believe that until a man can conquer all desire for personal aggrandizement, he is not in a position to be the greatest possible benefit to dentistry.

To those practitioners who are so situated that it is not practicable for them to unite in classes for post-graduate study, the only alternative is to adopt some system of study by which they can accomplish the most individually. And they must adhere to whatever system is marked out, for without systematic work little progress can be made.

Some one hour in each day can be laid aside for purely scientific study, and that hour invariably held sacred for that purpose. At first thought most practitioners will exclaim that they cannot possibly spare an hour every day for this purpose, but a little devotion to the interests of science will soon point out some one hour of the twenty-four which heretofore has not been occupied to the best advantage, and which "never will be missed." For the first month or two this study hour may at times be a little wearisome, but perseverance will soon overcome that, till at last it almost becomes a habit; and as the knowledge of scientific matters increases, the interest in the work will counteract the natural tendency to neglect that which we are not imperatively called upon to perform.

The line of study which each man is to pursue must be determined by himself, and in that department for which he has the best liking will he be the most certain of success. But before singling out any one department for minute study, he should gain a general fundamental knowledge of the basal principles relating to the whole of dental science. The main thing, however, is to get to work, and work faithfully, no matter how many are the discouragements. When we become a profession of workers and thinkers, we will become a profession of scientific advancement, and to that end let us all unite.

